# Dexamethasone administration during definitive radiation and temozolomide renders a poor prognosis in a retrospective analysis of newly diagnosed glioblastoma patients

**DOI:** 10.1186/s13014-015-0527-0

**Published:** 2015-10-31

**Authors:** Lisa B. E. Shields, Brent J. Shelton, Andrew J. Shearer, Li Chen, David A. Sun, Sarah Parsons, T. David Bourne, Renato LaRocca, Aaron C. Spalding

**Affiliations:** Norton Neuroscience Institute, Norton Healthcare, Louisville, KY USA; The Brain Tumor Center, Norton Healthcare, Louisville, KY USA; The Markey Cancer Center, University of Kentucky School of Medicine, Lexington, KY USA; The Norton Cancer Institute, Norton Healthcare, Louisville, KY USA; The Norton Cancer Institute Radiation Center, 676 S. Floyd St., Suite 100, Louisville, KY 40202 USA

**Keywords:** Radiation, Oncology, Dexamethasone, Bevacizumab, Temozolomide, Glioblastoma multiforme, Radiation, Survival

## Abstract

**Background:**

Dexamethasone (DXM) is commonly used in the management of cerebral edema in patients diagnosed with glioblastoma multiforme (GBM). Bevacizumab (BEV) is FDA-approved for the progression or recurrence of GBM but has not been shown to improve survival when given for newly diagnosed patients concurrently with radiation (RT) and temozolomide (TMZ). Both DXM and BEV reduce cerebral edema, however, DXM has been shown to induce cytokine cascades which could interfere with cytotoxic therapy. We investigated whether DXM would reduce survival of GBM patients in the setting of concurrent TMZ and BEV administration.

**Methods:**

We reviewed the treatment of all 73 patients with GBM who received definitive therapy at our institution from 2005 to 2013 with RT (60 Gy) delivered with concurrent daily TMZ (75 mg/m^2^). Of these, 34 patients also were treated with concurrent BEV (10 mg/kg every two weeks). Patients received adjuvant therapy (TMZ or TMZ/Bev) until either progression, discontinuation due to toxicity, or 12 months after radiation completion. All patients who had GBM progression with TMZ were offered BEV for salvage therapy, with 19 (56 %) receiving BEV.

**Results:**

With a median follow-up of 15.6 months, 67 (91.8 %) patients were deceased. The OS for the entire cohort was 15.9 months, while the PFS was 7.7 months. The extent of resection was a prognostic indicator for OS (*p* = .0044). The median survival following gross tumor resection (GTR) was 22.5 months, subtotal resection (STR) was 14.9 months, and biopsy was 12.1 months. The addition of BEV to TMZ with RT was borderline significantly associated with increased PFS (9.4 vs. 5.1 months, *p* = 0.0574) although was not significantly associated with OS (18.1 vs. 15.3 months respectively, *p* = 0.3064). In patients receiving TMZ, DXM use concurrent with RT was a poor prognostic indicator of both OS (12.7 vs. 22.6 months, *p* = 0.003) and PFS (3.6 vs. 8.4 months, *p* <0.0001). DXM did not reduce OS in patients who received TMZ and BEV concurrently with RT (22.9 vs 22.8 months, *p* = 0.4818). On multivariable analysis, DXM use predicted an unfavorable OS hazard ratio (HR) = 1.72, *p* = 0.045).

**Conclusions:**

Our results with TMZ, BEV, and RT are similar to previous studies in terms of PFS and OS. DXM use during RT with concurrent TMZ correlated with reduced OS and PFS unless BEV was administered.

## Introduction

The treatment of patients with GBM poses a challenge as the median patient survival rate after diagnosis is between 12 months with radiation alone and 14 ½ months with radiation and TMZ [1]. Several factors play a role in the prognosis of these patients, including age, preoperative performance status assessed by the Karnofsky Performance Scale, and extent of resection [2–5]. Resection of 98 % or more of the tumor has been shown to prolong survival in patients with GBM [4]. Furthermore, residual tumor enhancement by immediate postoperative MRI portends a significant reduction in survival as patients with a residual tumor postoperatively had a 6.595-times higher risk of death in comparison to patients without a residual tumor [2].

TMZ has been shown to improve OS and PFS when given concurrently with RT in newly diagnosed GBM patients [1, 6–9]. Additionally, adjuvant TMZ after completion of RT is a positive prognostic indicator of improved OS and PFS [1, 7–9]. As an alkylating agent, TMZ induces O^6^-methylguanine DNA lesions leading to apoptosis of tumor cells through mitochondrial activation of caspases in a Bcl-2 dependent mechanism [10]. *In vitro*, DXM protects GBM cells from TMZ-induced apoptosis by inhibition of caspase cleavage and alteration of bcl-2 levels [11]. Methylation status of the methyl-guanine methyl transferase gene (MGMT) promoter has been shown to be the strongest predictor for outcome and benefit from TMZ chemotherapy [8]. Tumors that do not express MGMT are more susceptible to chemotherapy with alkylating drugs, while patients with methylated MGMT promoter treated with TMZ and radiotherapy have long-term PFS [8].

DXM is commonly used in the management of GBM patients to treat intracranial edema and to control neurological symptoms, although there are no randomized trials of DXM investigating effects on survival [12]. While DXM may alleviate the negative symptoms that accompany GBM, patients are often encouraged to taper their use of DXM due to the side effects of hyperglycemia, osteoporosis, myopathy, weight gain, and immunosuppression [13]. DXM use may be reduced through administration of vascular endothelial growth factor (VEGF) inhibitors to minimize brain edema [12]. BEV is a monoclonal antibody that inhibits VEGF and may have a corticosteroid-sparing effect in patients with recurrent GBM [12].

We report here the OS and PFS of 73 newly diagnosed GBM patients with a focus on the effect of DXM with and without BEV. This cohort consisted of two initial regimens receiving identical RT: 1) concurrent and adjuvant TMZ, and 2) concurrent and adjuvant TMZ and BEV. This allowed us to study the interaction of DXM, TMZ, and BEV on the OS and PFS of newly diagnosed GBM patients.

## Methods

Under an institutional IRB-approved protocol and in compliance with the Helsinki Declaration, we retrospectively reviewed the treatment of 73 patients with GBM who received 30 fractions of simultaneous integrated boost IMRT delivered either with concurrent and adjuvant BEV and TMZ (combined, *n* = 34) or TMZ alone (standard, *n* = 39). This study included all patients at one center who were diagnosed with a GBM between 2005–2013. Our cohort spans an era prior to and after the reporting of RTOG 0825 and AVAGlio studies [14–16]. Patients prior to these studies received the combined arm while those after received standard therapy with BEV offered as salvage therapy in accordance with FDA approval and then possible BEV at relapse.

The extent of resection (gross total, subtotal, or biopsy only) was determined on 24-h postoperative MRI by a radiologist blinded to the neurosurgeon’s perspective. Preoperative and postoperative MRI scans were combined with the radiation planning CT scan. A MRI scan with gadolinium (GAD) was performed before initiation of adjuvant therapy and every subsequent three months. Radiation consisted of 30 identical fractions delivered once daily five times per week. The planning target volume prescribed 60 Gy (PTV60) received 2 Gy daily to the tumor bed or residual tumor delineated with T1 + GAD plus 1.5 cm margin while the planning target volume prescribed 54 Gy (PTV54) received 1.8 Gy to the T2 FLAIR +2.5 cm. The combined regimen consisted of concurrent TMZ (75 mg/m^2) daily and BEV (10 mg/kg every two weeks) administered during RT for six weeks. One month after completion of RT, adjuvant TMZ (150 mg/m^2 × 5 days) was delivered monthly with BEV (10 mg/kg) every two weeks until progression, toxicities, or 12 months total. The standard regimen involved RT to 60 Gy with concurrent TMZ (75 mg/m^2) given daily during RT for six weeks. One month after completion of RT, TMZ (150 mg/m^2 × 5 days) was delivered monthly for up to 12 months. At the time of disease progression, all patients were offered BEV infusion every three weeks as salvage therapy. A total of 19 patients received salvage BEV after progression on the standard arm.

All patients received DXM during the perioperative period which was weaned prior to the start of RT if possible. We categorized patients based on the use of DXM during RT for this analysis. Patients who could not taper off or who restarted DXM during RT were categorized as positive for steroid usage.

Progression free time refers to the interval between the diagnosis and progression. Progression was defined as: (1) New T1 and gadolinium enhancement; (2) T2 FLAIR progression; (3) new or worsening neurological symptoms; (4) a change in therapy; or (5) death. For patients receiving BEV, universally agreed criteria were used to determine progression [17]. The MRIs were independently reviewed by a neuroradiologist. The overall survival time was defined as the interval between diagnosis and death from any cause.

Descriptive analyses were conducted to estimate patient clinical and demographic characteristics. Kaplan-Meier analysis was performed to assess differences in event-free (progression and death) experience between groups using all patients as well as between groups using subsets of patients (eg. those only on TMZ). The log-rank test was invoked to assess statistical significance in univariable comparisons [18]. To adjust for potential covariate effects, Cox proportional hazards regression was used as the modeling paradigm [19]. Multivariate analysis was conducted using Cox regression modeling. The proportional hazards assumption was assessed both visually using plots of the observed standardized score process with 20 simulated realizations for each covariate included in the model as well as quantitatively using the Kolmogorov-type supremum test based on 1000 simulations [20].

## Results

### Whole cohort observations: univariable analysis

**Fig. 1:**
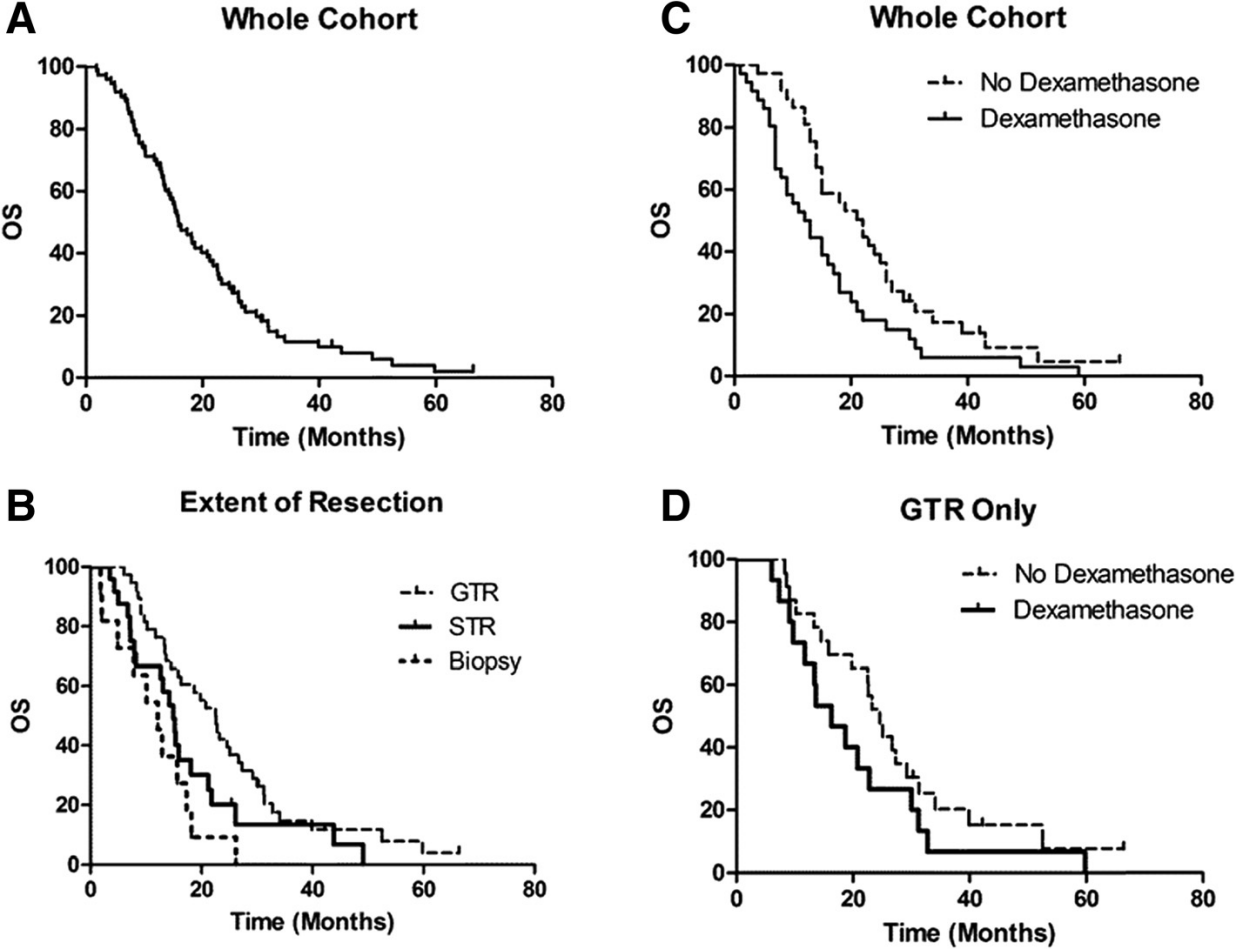
Kaplan-Meier curve shows the (**a**) OS of the whole cohort of newly diagnosed GBM patients after surgical resection, with a median of 15.9 months. **b** The extent of resection correlated with OS: GTR 22.6 months, STR 14.9 months, and biopsy 12.1 months. **c** DXM use concurrent with RT was a poor prognostic indicator of OS (median 12.7 vs. 22.5 months, *p* = 0.02 by log-rank test). **d** For patients who underwent GTR, those who were treated with DXM had a median survival of 16.3 months compared to 24.6 months without DXM (*p* = 0.15 by log-rank test)

A total of 73 patients were treated during this study with an age range at diagnosis between 28 and 79 years (median age 61 years) (Table 1). With a median follow-up of 15.6 months, 67 (91.8 %) patients were deceased. The average age at death was 60.5 years (age range 30–81 years). Forty-four patients (60 %) were male; 29 (40 %) were female. Two patients died before completion of RT due to progression after 6 Gy and 26 Gy, respectively. One patient developed a wound infection during radiation and received only 16 Gy. The median RT dose was 60 Gy. The majority (38; 52 %) of patients underwent a GTR, while 24 (33 %) underwent a STR and 11 (15 %) had a biopsy. Table 1 provides a description of this cohort of patients in terms of extent of resection, the concurrent regimen used, smoking, hypertension (HTN), body mass index (BMI), diabetes mellitus (DM), hyperlipidemia, and the use of DXM during RT. The OS for the whole cohort was 15.9 months (Fig. 1a), while the PFS was 7.7 months. The one-year and two-year survival was 70 and 31 %, respectively. The extent of resection was a prognostic indicator for OS (*p* = .0044) (Fig. 1b). The median survival following GTR was 22.6 months, STR was 14.9 months, and biopsy was 12.1 months. Patients who underwent a biopsy for GBM had a poor prognosis for OS. The addition of BEV to TMZ with RT was borderline significantly associated with increased PFS (9.4 vs. 5.1 months, *p* = 0.0574) although was not significantly associated with OS (18.1 vs. 15.3 months respectively, *p* = 0.3064).

**Table 1:** Population statistics of patients with newly diagnosed GBM

Population statistics	Category	Absolute	Percentage
Median age at diagnosis		61.0	
Average age at death		60.5	
Gender	M	44	60 %
	F	29	40 %
Extent of resection	GTR	38	52 %
	STR	24	33 %
	Biopsy	11	15 %
Median RT dose		6000	
Concurrent agents	Temozolomide alone	39	53 %
	Temozolomide and Bevacizumab	34	47 %
Smoker	No	43	60 %
	Yes	29	40 %
HTN	No	32	44 %
	Yes	41	56 %
BMI	Normal (<25)	17	23 %
	Overweight (25–30)	37	51 %
	Obese (>30)	19	26 %
DM	No	63	86 %
	Yes	10	14 %
Hyperlipidemia	No	46	63 %
	Yes	27	37 %
Dexamethasone during RT	No	37	51 %
	Yes	36	49 %

### Influence of dexamethasone: univariable analysis

DXM use concurrent with RT was a poor prognostic indicator of both OS (median 12.7 vs. 22.5 months, *p* = 0.02, Fig. 1c) and PFS (median 6.0 vs. 8.8 months, *p* = 0.002) in the whole cohort. Given the known significance of the extent of resection on survival, we analyzed the effect of DXM in patients with either a GTR (Fig. 1d) or STR/Biopsy. For patients who underwent GTR, those who were treated with DXM had a median survival of 16.3 months compared to 24.6 months without DXM (*p* = 0.15). Patients who underwent either STR or biopsy had nearly a two-fold reduction of median survival with DXM administration (8.2 months) compared to those without DXM (15.3 months) although statistical significance was not reached (*p* = 0.20).

**Fig. 2:**
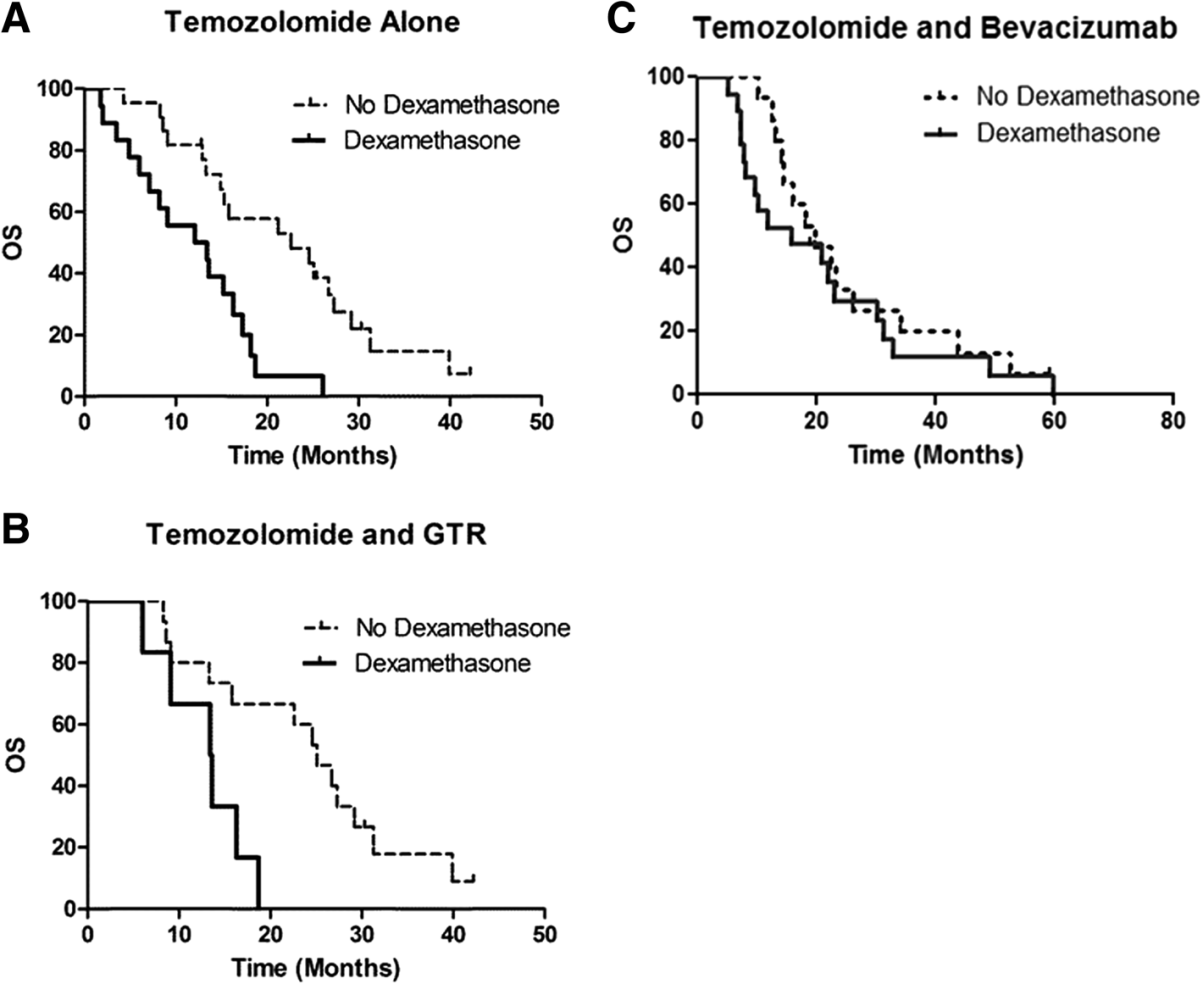
Kaplan-Meier curve of the (**a**) DXM use concurrent with RT was a poor prognostic indicator of OS in patients receiving TMZ (12.7 vs 22.6 months, *p* = 0.003 by log-rank test). **b** DXM use was a poor prognostic factor for OS in patients who underwent a GTR treated with TMZ alone (13.5 vs 25.1 months, *p* = 0.01 by log-rank test). **c** DXM did not reduce OS patients who received TMZ and BEV concurrent with RT (22.9 vs 22.8 months, *p* = 0.4818 by log-rank test)

Since BEV has been shown to reduce the use of DXM, we conducted additional analyses in patients who received TMZ only during RT without BEV. In patients receiving TMZ, DXM use concurrent with RT was a poor prognostic indicator of both OS (12.7 vs. 22.6 months, *p* = 0.003, Fig. 2a) and PFS (3.6 vs. 8.4 months, *p* < 0.0001). Even in patients who underwent a GTR treated with TMZ alone, DXM use was a poor prognostic factor for OS (13.5 vs 25.1 months, *p* = 0.01, Fig. 2b). Interestingly, DXM did not reduce OS in patients who received TMZ and BEV concurrently with RT (22.9 vs 22.8 months, *p* = 0.4818, Fig. 2c). DXM did not affect PFS in patients receiving concurrent BEV with TMZ (9.5 vs. 8.9 months, *p* = 0.6520).

### Multivariable analysis

Cox proportional hazards regression modeling was used to assess whether DXM administration continued to confer unfavorable time-to-event (progression and death) as seen in the univariable analyses. Two separate sets of multivariable models were run to supplement the respective univariable analyses. One set included all patients in the observation cohort. The second set included those administered only TMZ. In the first set of analyses (on the whole cohort), models for OS and PFS included the following covariate list: DXM, BEV, extent of resection, age at diagnosis, gender, XRT dosage, smoking status, and BMI. For the whole cohort analysis, significant predictors of unfavorable OS included patients with DXM (hazard ratio (HR) = 1.72, *p* = 0.045) and decreasing XRT dosage (a 1 sd decrease of 7.41 Gy results in HR = 1.66, *p* = 0.0035). Extent of resection was borderline significant (Wald chi-square *p* = 0.0617) for OS. Significant predictors of unfavorable PFS included patients with DXM (HR = 2.98, *p* = 0.0004) as well as patients without BEV (HR = 2.64, *p* = 0.0015). Results using only those administered TMZ were similar for OS to the whole cohort analysis: DXM use resulted in an increase in hazard of death (HR = 2.87, *p* = 0.0255) while decreasing XRT dosage by 1 sd (7.41 Gy) resulted in an increase in hazard of death (HR = 1.63, *p* = 0.0079). For PFS, DXM use was the only covariate found to be significantly associated with less favorable outcome (HR = 4.46, *p* = 0.0015).

There were no evidences for departure from the proportional hazards assumption for any of the covariates included in the models when inspecting the diagnostic plots of the observed standardized model process plotted over time. Additionally, all *p*-values associated with the Kolmogorov-type supremum test, which quantitatively tests for departures from proportional hazards, were not significant.

## Discussion

Several studies have investigated the combination of BEV and TMZ with RT in the treatment of newly diagnosed GBM [1, 21–27]. Our results compare favorably to these previous studies as the median OS, PFS, and one- and two-year survival data for patients with GBM who have been treated with a combination of RT and chemotherapy following initial resection are presented in Table 2. Our results with TMZ and RT are similar to previous studies in terms of PFS and OS. The OS for our whole cohort was 15.9 months, while the PFS was 7.4 months. The one-year and two-year survival in our study was 71 and 32 %, respectively. Concurrent BEV and TMZ with RT was significantly associated with increased PFS although was not significantly associated with OS. Our study also confirms the known literature that the extent of resection was a significant prognostic indicator for OS. The median survival following GTR was 22.5 months, STR was 14.93 months, and biopsy was 12.1 months.

**Table 2:** Survival for patients with GBM following radiation with biological agents

Study	Treatment	Median OS	Median PFS	One-year survival	Two-year survival
Current study (2015) (*n* = 73)	RT + BEV + TMZ (12 cycles)	15.9 months	7.7 months	70 %	31 %
Stupp [1] (*n* = 287)	RT + TMZ	14.6 months	6.9 months	61 %	27 %
Grossman [19] (*n* = 244)	RT + TMZ + novel agents	19.6 months	--	81 %	37 %
Lai [21] (*n* = 70)	RT + BEV + TMZ	19.6 months	13.6 months	--	--
Vredenburgh [24] (*n* = 75)	RT + BEV + TMZ + Irinotecan	21.2 months	14.2 months	78.7 %	44.9 %
Narayana [22] (*n* = 51)	RT + BEV + TMZ (six cycles)	23 months	13 months	85 %	43 %

*OS* Overall survival

*PFS* Progression-free survival

*RT* Radiotherapy

*BEV* Bevacizumab

*TMZ* Temozolomide

In Keth et al.’s study of 345 patients with newly diagnosed GBM, they demonstrated that GTR was a favorable prognostic factor for OS while the value of incomplete resection remained questionable [28]. They recommended biopsy only if GTR could not be safely performed as biopsy provided acceptable histological diagnosis and determination of MGMT promoter methylation status [28].

Two eagerly anticipated phase III trials (Avaglio and RTOG 0825) have focused on the upfront use of BEV with TMZ and RT in newly-diagnosed GBM [14–16, 29]. Both of these studies showed significantly prolonged PFS and similar adverse effects of BEV, however, the interim analysis of OS did not reach statistical significance [14–16, 30, 31]. The Avaglio study demonstrated maintenance of quality of life outcomes, while RTOG 0825 showed a decreased quality of life [14, 30, 31]. OS results from these two trials do not support routine upfront BEV. Questions remain whether BEV should be used to treat patients with recurrent GBM since it offers QOL and PFS benefits [31].

The Avaglio trial addressed the use of glucocorticoids in patients who were treated with BEV [29]. Of the patients who received DXM at baseline, DXM was discontinued in 66.3 % of patients who received BEV compared to 47.1 % of patients receiving placebo. Furthermore, of the patients who were not treated with DXM at baseline, the time to initiation of DXM was significantly longer with BEV than placebo [29]. Both of these observations support the hypothesis that BEV reduces cerebral edema to improve clinical symptoms.

DXM is commonly used in the treatment of GBM to manage cerebral edema and has anti-inflammatory properties [11, 32]. It is often prescribed at diagnosis to decrease tumor-associated vasogenic edema and improve symptoms [13]. DXM is often continued after biopsy or resection to reduce post-operative edema and then continued during RT to decrease radiation-associated edema [13]. DXM is occasionally utilized concurrently with TMZ and RT after the initial surgical resection of a GBM. It has been shown that DXM works as an antagonist on TMZ-induced apoptosis in human glioblastoma T98G cells, suggesting that the combination of TMZ and DXM may be counteractive in treating GBM [11].

Due to the toxicities associated with DXM, including hyperglycemia, myopathy, osteoporosis, and immunosuppression, many patients with GBM decrease or discontinue their DXM use early in their management of GBM [12, 13]. Contrarily, other patients may necessitate an increase in DXM if they experience focal neurologic symptoms (hemiparesis, visual field deficit, or aphasia) or global symptoms (headache, nausea, or decreased appetite) [13].

Cerebral edema is a common side effect of chemoradiotherapy for GBM, necessitating glucocorticoid management [33]. Substantial deregulation of blood glucose levels may ensue. In their study of 106 GBM patients, Mayer et al. determined that one or more deregulated blood glucose values > 10 mM was associated with a reduction in median OS from 16.7 to 8.8 months [33]. In addition, a significantly poorer OS was found in patients with hyperglycemia who underwent complete tumor resection.

Several reports have demonstrated DXM dependency during RT as an independent poor predictor of survival in GBM [32, 34, 35]. In their study of 173 patients with malignant gliomas, Watne et al. reported that patients who were corticosteroid-dependent after craniotomy had a 1.9 relative death risk as compared to patients who were off steroids post-operatively [35]. They did not examine whether the increased relative risk of death associated with corticosteroid use was influenced by the extent of resection. Michaelsen et al. reported that the use of corticosteroid therapy was significantly correlated with patient survival and disease progression in their study of 225 GBM patients [34]. Corticosteroid therapy at TMZ and RT initiation had a significant negative impact on OS (*p* < 0.0001). This study examined the interaction of age with corticosteroid use but did not investigate the interaction of extent of resection and corticosteroid use. Patients with a GTR would be expected to have the longest OS. These data together demonstrated that DXM administration produces shorter overall survival even without measurable contrast-enhancing tumor, suggesting the effect is independent of the extent of resection. We found by both univariate and multivariable analysis that DXM use correlated with decreased OS. Thus, our data both reinforce previous work as well as expand the conclusions to include the extent of resection along with DXM use.

Gorlia et al. analyzed the data from 300 patients with recurrent GBM in eight phase I or II trials conducted by the EORTC Brain Tumor Group [36]. A total of 138 patients received TMZ and RT followed by TMZ as first-line therapy, while 158 patients received RT alone or with another chemotherapy; four were treated without previous radiotherapy [36]. They demonstrated that patients who received TMZ and RT followed by TMZ were significantly less often treated with baseline steroids (57 % vs 73 %, *p* = 0.004). They concluded that patients treated with steroids at baseline had a shorter OS. Furthermore, they suggested that the use of anti-angiogenic therapies may change the prognostic potential of certain factors, such as BEV administration may reduce the detrimental effect of the need for steroids, at least on PFS [36].

Vredenburgh et al. investigated the effect of corticosteroid use in GBM patients at first or second recurrence treated with BEV in the BRAIN study [12]. The BRAIN study was a phase II, multicenter, randomized trial of BEV alone or concurrent with irinotecan (CPT-11) in patients with recurrent GBM. The authors reported that the majority of patients who had an objective response or PFS > 6 months experience corticosteroid dose reduction. They suggested that BEV may have a corticosteroid-sparing effect in patients with recurrent GBM and that a reduction in corticosteroids may positively affect patient QOL [12].

Our present study concurs with previous studies that DXM use concurrent with RT is a significantly poor prognostic indicator of both PFS and OS in patients with GBM. Our study is the first in the literature, to our knowledge, that investigated upfront DXM use concurrently with RT, TMZ, and BEV. In patients who received TMZ, DXM use concurrent with RT was a poor prognostic indicator of both OS and PFS. However, DXM did not reduce OS or affect PFS in patients who received TMZ and BEV concurrently with RT. In addition, of all of the patients treated with DXM and RT, the OS was significantly shorter in patients who only received TMZ compared to those who received both TMZ and BEV. Our results suggest that it is not recommended to treat GBM patients with upfront DXM or with DXM concurrent with TMZ. However, if DXM is necessary due to the negative side effects of GBM such as headaches, then the administration of BEV concurrent with DXM may be beneficial. We have shown that DXM use during RT with concurrent TMZ correlated with reduced OS and PFS unless BEV was administered.

## Conclusion

The combination therapy of TMZ, BEV, and RT was safe and well tolerated in this study. Similar to previous reports in the literature, RT with concurrent BEV and TMZ was significantly associated with increased PFS but not OS. The present study demonstrates that DMX use concurrent with RT and TMZ was a poor prognostic indicator of both OS and PFS. Contrarily, DMX did not reduce OS in patients who received TMZ and BEV concurrently with RT. Upfront use of BEV may prove beneficial in GBM patients who require DXM use and are treated with concurrent TMZ and RT.
